# Post-intensive care screening: French translation and validation of the Healthy Aging Brain Care-Monitor, hybrid version

**DOI:** 10.1186/s12955-022-01967-1

**Published:** 2022-04-02

**Authors:** Geoffrey Horlait, Charlotte Beaudart, Laurine Bougard, Stephen Bornheim, Camille Colson, Benoit Misset, Olivier Bruyère, Malaz Boustani, Anne-Françoise Rousseau

**Affiliations:** 1 Intensive Care Department, CHU UCL Namur, Université Catholique de Louvain, Louvain-la-Neuve, Belgium; 2 Department of Physical Medicine and Rehabilitation, University Hospital, University of Liège, Liège, Belgium; 3 Intensive Care Department and Burn Center, University Hospital, Sart-Tilman B35, Avenue de l’Hôpital 1, 4000 Liège, Belgium; 4 Division of Public Health, Epidemiology and Health Economics, World Health Organization Collaborating Centre for Public Health Aspects of Musculoskeletal Health and Ageing, University of Liège, Liège, Belgium; 5 Sandra Eskenazi Center for Brain Care Innovation, Eskenazi Health, Indianapolis, IN USA

**Keywords:** Critical illness, Survivors, Post-intensive care syndrome, Healthy Aging Brain Care Monitor, Functional capacity, Anxiety, Depression, Cognitive disorders, Health-related quality of life

## Abstract

**Background:**

The Healthy Aging Brain Care-Monitor (HABC-M) questionnaires (self-reported version and caregiver version) have been validated for post-intensive care syndrome (PICS) detection in patients surviving a stay in the intensive care unit (ICU). Their authors have also developed a hybrid version (HABC-M-HV) suited to the daily needs of their post-ICU follow-up clinic. The objectives of the present cross-sectional observational study were to translate the HABC-M-HV questionnaire into French (HABC-M-HV-F) according to international guidelines and to test its measurement properties.

**Methods:**

The HABC-M-HV was translated according to international guidelines. The measurement performances of the questionnaire were tested using internal consistency, test–retest reliability, Standard Error of Measurement (SEM) and Smallest Detectable Change (SDC) calculation, floor and ceiling effect measurement and construct validity.

**Results:**

The validation study included 51 ICU survivors (27.5% women, 63 [55–71] years old). The questionnaire was administered by phone. The internal consistency was very good (Cronbach’s alpha coefficient 0.79). The intra- and inter-examinator reliabilities were excellent (Intraclass Coefficient Correlation = 0.99 and 0.97, respectively). The SEM was 0.62 and the SDC was 1.72. No floor or ceiling effects were observed. The convergent validity was almost entirely confirmed with 71.4% of our hypothesis confirmed.

**Conclusion:**

The HABC-M-HV-F has been shown to be a valid and reliable tool for PICS screening and follow-up in French-speaking ICU survivors. A remote administration by phone was feasible.

*Trial registration*: Not applicable.

**Supplementary Information:**

The online version contains supplementary material available at 10.1186/s12955-022-01967-1.

## Key points

- A new hybrid version of the HABC-M is now available in French for post-intensive care syndrome detection.
- The questionnaire demonstrates good validity and good reliability.
- The HABC-M-HV-F can be remotely administered by phone.

## Background

Patients surviving a stay in intensive care unit (ICU) may experience mid- and long-term morbidities related to the critical illness, the treatment and organ support received, and the unique ICU environment. These new or worsening disorders have been labelled as “post-intensive care syndrome” (PICS). This term generally refers to muscle weakness and reduced autonomy for daily activities, mental disorders (anxiety, depression, post-traumatic stress syndrome) and neurocognitive impairments, that can all negatively impact survivors ‘quality of life [1].

PICS and its related needs can be addressed either by primary care physicians and healthcare providers or by dedicated ICU follow-up clinics that are increasingly available worldwide [2]. The first step of the post-ICU trajectory is to measure the PICS related outcomes. For a given outcome, there are often different ways of measuring it. Some core outcome sets have been created to rationalize the heterogeneity of measures that can be used to assess survivors [3]. A face-to-face consultation allows clinicians to measure the different PICS outcomes using validated questionnaires and tests. Yet, the delivery and provision of post-ICU follow-up is highly variable between centres and geographical locations, in terms of resources allocated and supports provided. Telemedecine is an alternative to face-to-face consultations, booming since the COVID-19 pandemics [4]. However, with such a medical practice, some outcomes are assessed more superficially: some questionnaires are too complex or too long to be remotely administered, and some tests require direct contact with the patient.

The Healthy Aging Brain Care Monitor (HABC-M) is a questionnaire that was initially developed to address the needs of primary care providers for a practical, multidomain instrument assessing cognitive, functional and psychological symptoms of elderly patients. Two versions were developed and validated: The Caregiver Report Version (31-item questionnaire) relied on the observations and perceptions of the patient’s informal caregiver [5], while the Self-Report Version (27-item questionnaire) was utilized to collect information directly from the patient [6]. Both versions have recently been validated for PICS screening [7, 8]. During the implementation of these tools in daily practice, their authors used an agile implementation process [9] to meet the changing local needs and to deal with the local context. During this process, a hybrid version of the HABC-M questionnaire (combining 26 questions of the Self Report version and the 4 questions on quality of life, specific to the Caregiver Report) appeared as a useful alternative (Additional file 1: Figure S1). This 30-item hybrid version includes a cognitive subscale consisting of 8 questions on memory, orientation and judgment, a functional subscale consisting of 8 questions on activities of daily living, a psychological subscale consisting of 10 questions on anxiety, depression and psychosis and a final section of 4 questions investigating perceived quality of life. Each question is rated from 0 to 3, based on patient’s perception of the frequency of symptoms, and total score thus ranges from 0 to 90. In view of its composition, this version was thought to be particularly adapted for PICS screening. However, the hybrid version of the HABC-M questionnaire (HABC-M-HV) has not yet been validated in its English version.

The objective of the present study was to translate the HABC-M-HV into French according to international guidelines and to evaluate the main measurement properties of this new version. This would be the first validated version of this tool.

## Methods

### French translation of the HABC-M-HV

**Fig. 1:**
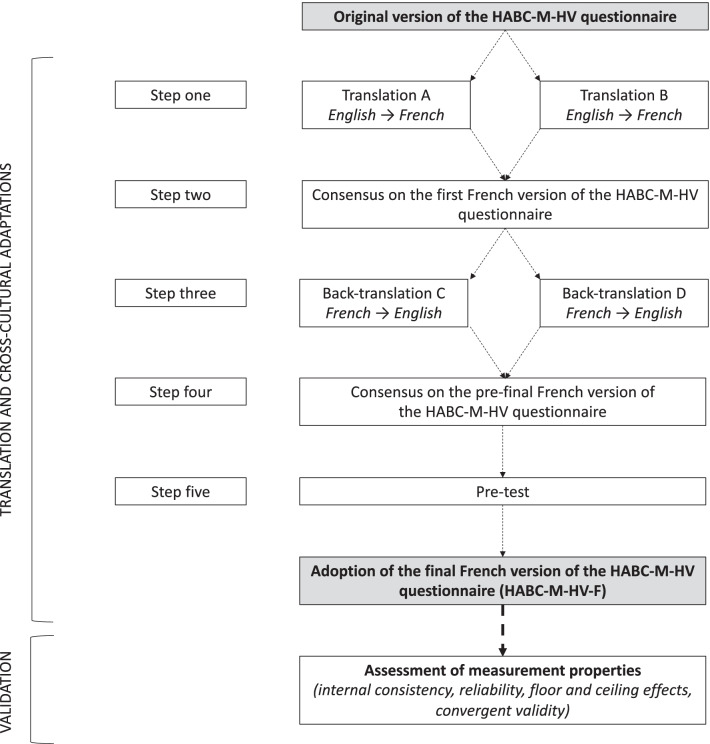
Flow chart of the HABC-M-HV translation and validation

The questionnaire was translated following a five-stage validated method for the translation and cross-cultural adaptation of questionnaires [10] (Fig. 1). Firstly, two bilingual experts with French as their mother tongue independently translated the HABC-M-HV questionnaire from English to French. Secondly, the two translators summarized the results of their two translations and agreed on a first consensual translated French version of the HABC-M-HV. Thirdly, two other bilingual translators with English as their mother tongue, blinded to the original version of the HABC-M-HV, independently translated the French HABC-M-HV back into English. Fourthly, a committee composed of the four translators (including an intensive care physician) and a French language specialist met to review all the translations of the questionnaire and develop what would be considered the prefinal version of the translation for field testing. They ensured equivalence between the source and target version in four areas: semantic, idiomatic, experiential and conceptual equivalences. Finally, the prefinal French version of the HABC-M-HV questionnaire (HABC-M-HV-F) was preliminary tested on a sample of 10 participants working in ICU to assess its understanding and clarity. Following this last step and considering potential last changes to increase understandability of the questionnaire, the HABC-M-HV-F was considered as the final one.

### Measurement properties of the HABC-M-HV-F

The study of the measurement properties was performed considering the principles of the Consensus-based Standards for the Selection of Health Status Measurement Instruments (COSMIN) recommendations [11].

#### Ethics

Ethic approval of the study protocol (Local Ref: 2020/257) was provided by the local ethics committee of our University Hospital of Liège (Chairperson: Pr Vincent Seutin) on 28 July 2020.

#### Participants

A convenience sample with French-speaking ICU survivors was recruited in our post- intensive care follow-up clinic in Liège, Belgium. Exclusion criteria were a documented history of dementia, deafness or hearing loss, speech disorders and refusal to participate. Oral informed consent was obtained before enrolment. HABC-M-HV-F was administered by phone to test the feasibility of a remote administration. Thereby, it was administered the day after the face-to-face consultation at our follow-up clinic.

#### Internal consistency

Internal consistency is defined as the degree of homogeneity across items [11] and is measured with the Cronbach’s alpha coefficient. This coefficient ranges from 0 to 1 with higher values representative of higher internal consistency. It has been recognized that a value between 0.7 and 0.9 reflects a good internal consistency of the scale without significant risk of redundancy of items [11, 12]. To measure internal consistency, we first measured a global alpha coefficient for the HABC-M-HV-F questionnaire. We also assessed the impact of deleting each domain on the total internal consistency. Finally, we measured the correlation of each domain with the global score of the HABC-M-HV-F. Spearman (rs) correlation was considered weak if < 0.2, between 0.2–0.4 as acceptable, between 0.4–0.6 as good, and > 0.6 as strong [13].

#### Test–retest reliability

Test–retest reliability reflects the capacity of a questionnaire to be reliable and to produce the same scores for repeated measurements in participants whose health status has not changed. To measure test–retest reliability, all patients were invited to answer the questionnaire for a second time on the day following the first administration. This short delay was intended to limit the risk of health status changes, that can be frequent in the post-ICU period. Patients were called either by the same investigator or by the co-investigator, to test both intra- and inter-examinator reliability. Test–retest reliably was assessed with the intraclass coefficient correlation (ICC) and its 95% confidence interval (95% CI). We used a two-way mixed method for absolute agreement. ICC improves as it approaches 1 and the reliability is considered as acceptable with an ICC of 0.7 [14].

The standard error of measurement (SEM) and smallest detectable change (SDC) of the HABC-M-HV-F were also calculated. The SEM, which provides a range around the observed value in which the theoretical true value can be found, was measured by dividing the standard deviation of the difference between the test and the retest by the square root of 2. The SDC, which indicates the amount of change that needs to be measured to be sure that the change measured is real and not due to a potential measurement error, was measured using the following formula: 1.96 ∗ SEM ∗ √2 [15].

#### Floor and ceiling effects

Floor and ceiling effects were considered to be present when more than 15% of the population obtained a maximum score (ceiling effect) or a minimum score (floor effect) [16]. When floor or ceiling effects are present, participants with the minimum or maximum score cannot be distinguished from one another, reducing the discriminative power of the questionnaire.

#### Construct validity

Construct validity ensures that the scale truly measures what it is supposed to measure. For this purpose, the convergent validity was measured using the correlation between each section of the questionnaire and related validated questionnaires. These questionnaires are routinely administered during the face-to-face consultation at our post-ICU follow-up clinic. Section 1 of the HABC-M-HV-F (exploring cognition) was correlated to the Montreal Cognitive Assessment tool (MoCA). This test examines visuospatial abilities, executive function, attention/working memory, episodic memory, and language. The MoCA total score was used for analysis: it ranges from 0 to 30, the lower scores indicating worse cognitive performances [17]. Section 2 of the HABC-M-HV-F (exploring functional status) was correlated to Barthel index, a questionnaire measuring functional status and dependency. It consists of 10 subheadings, namely feeding, bathing, grooming, dressing, bladder control, bowel control, toilet use, chair–bed transfer, mobility and stair climbing [18]. Scoring ranges from 0–100: a score of 100 is defined as being capable of complete self-care. Section 3 of the HABC-M-HV-F was correlated to two different questionnaires exploring mental health status: the Hospital Anxiety and Depression scale (HADS) and the Impact of Event Scale-Revised (IES-R). The HADS consists of two 7-item subscales evaluating symptoms of depression (seven items-HADS-D subscale) and symptoms of anxiety (seven items—HADS-A subscale) [19]. Scoring ranges from 0 to 21 on either subscale: the standard cutoff threshold value of > 7 out of 21 on either subscale was used to define a borderline status (score 8–10) or clinically significant status (score 11–21) of depression or anxiety, respectively. The IES-R is a 22-item tool that detects symptoms indicating a post-traumatic stress disorder [20]. It measures the severity of the three categories of post-traumatic stress disorder symptoms: avoidance, intrusion and hyperarousal symptoms. Scores higher than 33/88 indicate severe psychological impact of the traumatic event. Finally, Sect. 4 of the HABC-M-HV-F exploring health-related quality of life (HR-QoL) was correlated to the EQ-5D-3L. This tool is comprised of two sections: a five-question descriptive component which explores five dimensions: mobility, self-care, usual activities, pain/discomfort and anxiety/depression. Each question has three possible answers, rated from 1 to 3 (i.e. no, some or extreme problems). The second section is a visual analogue scale (EQ VAS) of HRQoL.

We made prior hypotheses and assumed that significant moderate to strong correlations would be observed between the HABC-M-HV-F domains and their related reference questionnaires. The construct validity was considered good if at least 75% of our hypotheses were confirmed by analyses [16].

#### Statistical analysis

The data was processed using the SPSS Statistics 24 (IBM Corporation, Armonk, NY) software package. The results were considered statistically significant at the 5% critical level. The normality of the variables was checked by examining the histogram, the quantile–quantile plot, the Shapiro- Wilk test, and the difference between the mean and the median values. As the majority of datasets did not pass the normality test, results were expressed as medians with interquartile ranges [P25 and P75] for quantitative parameters. Qualitative variables were described by absolute and relative (%) frequencies. The correlation between two quantitative variables was assessed using the Spearman coefficient (r_s_).

## Results

### French translation of the HABC-M-HV

The 30 items of the HABC-M-HV were translated without any difficulties. The pretest revealed no issues with understanding the French-translated version of the HABC-M-HV. The HABC-M-HV-F is available in Additional file 2: Figure S2.

### Measurement properties of the HABC-M-HV-F

#### Population

A total of 51 patients were recruited between February and September 2021. Descriptive characteristics of the included subjects are detailed in Table 1. Scores for the HABC-M-HV-F and the reference questionnaires are detailed in Table 2.

**Table 1:** Patients characteristics

Data	n = 51
Age, y	63 [55–71]
Male, n (%)	37 (72.5)
Admission type, n (%)	
Medical	35 (68.6)
Surgical	16 (31.4)
Admission failure, n (%)	
Cardiovascular	11 (21.6)
Pulmonary	29 (56.9)
Neurologic	5 (9.8)
Other	6 (11.7)
SOFA at admission	5 [4–7]
SAPS II	36 [27–53]
Mechanical ventilation, n (%)	29 (56.9)

*ICU* intensive care unit, *LOS* length of stay, *SAPS II* simplified acute physiology score, *SOFA* sequential organ failure assessment

**Table 2:** Scores for the HABC-M-HV-F and reference questionnaires

Questionnaire (and range of possible score)	Observed score, median [P25 and P75]	Observed minimum and maximum
HABC-M-HV-F		
Total (0–90)	11 [6–16]	0–60
Section 1 (0–24)	1 [0–4]	0–20
Section 2 (0–24)	3 [0–4]	0–18
Section 3 (0–30)	3 [0–5]	0–20
Section 4 (0–12)	3 [1–4]	0–12
MoCA (0–30)	27 [25–28]	10–30
Barthel index (0–100)	100 [100–100]	40–100
HADS		
HADS-A (0–21)	2 [0–5]	0–17
HADS-D (0–21)	1 [0–5]	0–14
IES-R (0–88)	6 [4–16]	0–64
EQ-5D-3L		
Score (3–15)	7 [5.75–8]	5–12
VAS (0–100)	70 [63.75–80]	10–100

#### Internal Consistency

A total Cronbach’s alpha of 0.79 has been found, revealing a very good internal consistency. Deleting Sect. 1 slightly reduced internal consistency, with a Cronbach’s alpha lowering to 0.67. However, the highest Cronbach’s alpha of 0.87 was found when deleting Sect. 2. All items showed strong and significant correlations with the total score of the HABC-M-HV-F (all r_s_ ≥ 0.6) (Table 3).

**Table 3:** Results of internal consistency and reliability

HABC-M-HV-F	Internal consistency			Reliability			
	Cronbach’s alpha if one section deleted (n = 51)	Correlation with total score (n = 51)		Test–retest reliability (n = 26)		Inter-examinator reliability (n = 23)	
		r_s_	*p* value	ICC	95% CI	ICC	95% CI
Section 1	0.67	0.69	< 0.001	0.97	0.93–0.98	0.83	0.64–0.93
Section 2	0.87	0.78	< 0.001	1		0.92	0.82–0.97
Section 3	0.71	0.65	< 0.001	0.98	0.96–0.99	0.91	0.79–0.96
Section 4	0.70	0.72	< 0.001	0.99	0.98–0.99	0.91	0.79–0.96
Total				0.99	0.98–0.996	0.97	0.93–0.99

*CI* confidence interval, *HABC-M-HV-F* Healthy Aging Brain Care-Monitor-Hybrid Version-French, *ICC* interclass correlation

#### Test–retest reliability

The intra-examinator reliability was tested in 26 patients and the inter-examinator reliability was tested in 23 patients. Total scores of the HABC-M-HV-F were very similar between test and retest with the same examinator, respectively 9 [3.75–15.5] and 8.5 [3.75–16.25]. The HABC-M-HV-F had an excellent test–retest reliability with an ICC value of 0.99 (95% CI 0.98–0.996) for the total score. All ICC values for individual sections were excellent as well (Table 3). Total scores of the HABC-M-HV-F were very similar between test and retest with two examinators, respectively 12 [6–24.5] and 12 [4.-23.5]. The inter-examinator reliability was excellent too: ICC value for the total score was 0.97 (95% CI 0.93 to 0.99) and ICC for the four different sections were in similar ranges (Table 3). The scores obtained in the 26 first patients were not statistically different from the scores obtained in the 25 other patients (*p* = 0.336 for test and *p* = 0.434 for retest).

The standard error of measurement was calculated to be 0.62 points and the smallest detectable change was 1.72 points.

#### Floor and ceiling effects

Four patients (7.8%) obtained a score of 0 on the HABC-M-HV-F questionnaire, while no patients obtained the maximum score. Therefore, neither floor nor ceiling effect was observed.

#### Construct validity

We validated 71.4% (5/7) of our hypothesis on convergent validity. Moderate significant correlations were found between Sect. 2 of the HABC-M-HV-F questionnaire and the Barthel index, between Sect. 3 and the HADS-A and HADS-D questionnaires, and between Sect. 4 and the EQ-5D-3L questionnaire. However, no significant correlation was found neither between Sect. 1 and the MoCA tool nor between Sect. 3 and the IES-R questionnaire (Table 4).

**Table 4:** Results of the convergent validity measurement

	r_s_	*p* value	Hypothesis validated?
MoCA versus section 1 score	0.052	0.72	No
Barthel versus section 2 score	− 0.38	0.006	Yes
HADS-A versus section 3 score	0.46	0.001	Yes
HADS-D versus section 3 score	0.45	0.001	Yes
IES-R versus section 3 score	0.17	0.25	No
EQ-5D-3L score versus section 4 score	0.45	0.001	Yes
EQ-5D VAS versus section 4 score	− 0.38	0.007	Yes

## Discussion

The present study allowed the development of a French translated version of the HABC-M-HV. Good measurement properties were observed during the validation process in ICU survivors. Its administration by phone was feasible. The observed scores were similar to those reported in a recent study including COVID-19 ICU survivors and assessed using the Self-Reported version of the HABC-M [21].

Internal consistency was considered good (Cronbach’s alpha of 0.79). Deleting Sect. 2 resulted in an improvement of Cronbach’s alpha to 0.87. However, the difference is not significant, leading us to conclude that this section does not have a negative impact on the questionnaire. Moreover, this section provides valuable information on one of 3 main domains of PICS according to its princeps definition. From another point of view, all sections were strongly correlated with the total score of the questionnaire. Overall, our data supports the retention of all sections of the HABC-M-HV-F.

No floor or ceiling effects were detected. This measurement property is important regarding the discriminative power of the questionnaire. For example, a maximum score would not allow any improvement in the questionnaire to be seen following any type of intervention.

Both intra-and inter-examinator reliability were excellent. This means that the HABC-M-HV-F is suitable for a longitudinal follow-up, regardless of the setting and the examinator. The SDC was measured at 1.72 points, which means that a patient would have to change by at least this amount before considering an improvement or a deterioration of their condition. Regarding SEM, we found a value of 0.62, meaning that we can be 68% confident (± 1SEM) that the “true” score of a patient can be found between − 0.62 and + 0.62 points from the observed score. The smaller the SEM is, the more confident we can be in the measured score.

The convergent validity was almost entirely confirmed with 71.4% of our hypothesis confirmed. We did not confirm a correlation between MoCA test and the cognitive subscale of the HABS-M-HV-F. The MoCA probes several cognitive domains including executive functioning, immediate and delayed memory, working memory, visuospatial abilities, and language. These domains cannot be objectively explored with a restricted number of closed questions such as those presented in the HABC-M-HV-F. Similarly, we did not confirm a correlation between the IES-R questionnaire and the psychological subscale of the HABS-M-HV-F. The latter does not explore specifically the multiple warning signs of post-traumatic stress disorder. However, the HABC-M-HV-F should not be considered as a diagnostic tool, but rather as a screening tool. From this point of view, the HABC-M-HV-F meets this requirement. If positive or worsened over time, it should trigger further precise investigations to thoroughly explore the impaired domains.

Measurement properties of the HABC-M-HV-F seem consistent with those obtained from the original versions of the HABC-M in PICS context [7, 8]. For these versions, authors observed similar internal consistency, and similar findings regarding convergent validity. However, to the best of our knowledge, intra or inter-examinator reliability was not assessed. No other translations of the HABC-M or the HABC-M-HV have been published. Nonetheless, the hybrid version has the advantage of including an evaluation of the patient’s perceived quality of life. This widens the spectrum of PICS’ features detection. As tested in the present study, the other advantage of this version is to be suitable for a remote administration, by phone for example. This is very interesting in the context of post-ICU follow-up. Attendance to follow-up consultation by ICU survivors may often be limited by physical disabilities, transportation difficulties related to reduced financial resources or social isolation, admission in nursing homes, overbooked agendas by rehabilitation sessions, hospital readmission or lack of appreciation of the benefits of such a consultation. In these contexts, telemedecine is an advantageous alternative to face-to-face consultation, but requires adapted screening tools. The HABC-M-HV-F has been shown to be easily administered by phone and could be useful for remote post-ICU follow-up.

The main strength of the study is the rigorous methodology employed for the translation and the validation of the HABC-M-HV-F questionnaire. Moreover, the convergent validity was explored using validated questionnaires that are frequently reported in publications about PICS and that are part of core outcome sets recommended to evaluate some categories of ICU survivors [3]. This study has also some limitations. Firstly, because of the cross-sectional design of the study, we were unable to measure responsiveness of the HABC-M-HV-F in our population. To a greater extent, further studies should aim to define the clinimetric properties of the present hybrid version, giving a more clinical insight to the HABC-M-HV as a measure of patient-reported outcomes [22]. Secondly, the sample size of participants was limited. However, it reached 51 participants, just higher than the minimal recommended sample size for measurement properties of health status questionnaires [16]. Thirdly, the retest was scheduled the after the test, aiming to limit the risk of health status changes. At the opposite, it could have induced a recall bias, as participants may have still remembered the response they gave the day before. However, we assume the number of answer possibilities for each of the 30 questions could have limited this risk.

## Conclusion

A new hybrid version of the HABC-M is now available in French. The questionnaire demonstrates good validity and good reliability. The HABC-M-HV-F can be used with confidence for PICS screening and follow-up and can be remotely administered by phone if the patient is unable to attend a consultation. This questionnaire can be used as a standardized method for research or clinical purposes throughout the post-ICU trajectory.

## Supplementary Information

**Additional file 1. Figure S1:** The HABC-M-HV (English)

**Additional file 2. Figure S2:** The HABC-M-HV-F questionnaire

## Data Availability

The datasets used and/or analyzed during the current study are available from the corresponding author on reasonable request.

## Abbreviations

HABC-M: Healthy Aging Brain Care-Monitoring
HABC-M-HV: Healthy Aging Brain Care-Monitoring-Hybrid version
HABC-M-HV-F: Healthy Aging Brain Care-Monitoring-Hybrid version in French
HADS: Hospital Anxiety and Depression scale
HRQoL: Health-related quality of life
ICC: Interclass coefficient correlation
ICU: Intensive care unit
IES-R: Impact of event scale-revised
LOS: Length of stay
MoCA: Montreal cognitive assessment
PICS: Post-intensive care syndrome
SDC: Smallest detectable change
SEM: Standard error of measurement
VAS: Visual analogic scale
