# Supplementary material for: Post-intensive care screening: French translation and validation of the Healthy Aging Brain Care-Monitor, hybrid version
Source: Health Qual Life Outcomes. 2022 Apr 2;20:59. doi: 10.1186/s12955-022-01967-1 (PMC8976274; doi:10.1186/s12955-022-01967-1)
Supplement: Supplementary file 1 — Additional file 1. Figure S1: The HABC-M-HV (English) [file 12955_2022_1967_MOESM1_ESM.pdf]

ESKENAZI HEALTH

Indianapolis, Indiana

Rev. 7/13

HABC MONITOR: SR VERSION - PAGE 2 OF 2

	Over the past two weeks, how often did you have problems with: (Use √ to indicate your answer.)	Not at all (0-1 day) 0 Points	Several Days (2-6 days) 1 Point	More than half the days (7-11 days) 2 Points	Almost Daily (12-14 days) 3 Points
SECTION 1	Judgement or decision-making				
	Less interest of/or pleasure in doing things, hobbies or activities				
	Repeating the same things over and over, such as questions or stories				
	Learning how to use a tool, appliance, or gadget				
	Forgetting the correct month or year				
	Handling complicated financial affairs: balancing checkbook, income taxes & paying bills				
	Remembering appointments				
	Thinking or memory				
SECTION 2	Planning, preparing, or serving meals				
	Taking medications in the right dose at the right time				
	Walking or physical ambulation				
	Bathing				
	Shopping for personal items like groceries				
	Driving				
	Falling or tripping				
	Housework or household chores				
SECTION 3	Feeling down, depressed or hopeless				
	Feeling lonely				
	Resisting help from others or getting agitated				
	Feeling anxious, nervous, tense, fearful or panic				
	Believing others are stealing from you or planning to harm you				
	Hearing voices, seeing things or talking to people who are not there				
	Poor appetite or overeating				
	Falling asleep, staying asleep, or sleeping too much				
	Acting impulsively, without thinking through the consequences of your actions				
	Wandering, pacing, or doing things repeatedly				
SECTION 4	**Over the past two weeks, how often did you have problems with:**				
	**Your** quality of life				
	**Your** financial future				
	**Your** mental health				
	**Your** physical health				
	**Column Totals**				
	**Total Score**				

1153D2 OF 2
