# Supplementary material for: Post-intensive care screening: French translation and validation of the Healthy Aging Brain Care-Monitor, hybrid version
Source: Health Qual Life Outcomes. 2022 Apr 2;20:59. doi: 10.1186/s12955-022-01967-1 (PMC8976274; doi:10.1186/s12955-022-01967-1)
Supplement: Supplementary file 2 — Additional file 2. Figure S2: The HABC-M-HV-F questionnaire [file 12955_2022_1967_MOESM2_ESM.pdf]

	Healthy Aging Brain Care-Monitor-Hybrid version - French (HABC-M-HV-F)<br>*Quand vous complètez cette évaluation, merci de garder à l'esprit que votre réponse doit être votre première réaction à la question. Il n'y a pas de définition formelle pour les symptômes que l'on vous demande d'évaluer. C'est pourquoi des exemples sont parfois inclus.*	**Pas du tout** (pendant 0 ou 1 jour)	**Durant quelques jours** (pendant 2 à6 jours)	**Plus de la moitié du temps** (pendant 7 à 11 jours)	**Tous les jours ou presque** (12- 14 jours)
SECTION 1	***Au cours des deux dernières semaines, avez-vous eu des problèmes pour***				
	Prendre une décision, porter un jugement	0	1	2	3
	Apprendre à utiliser un outil, un appareil, un accessoire ou un gadget	0	1	2	3
	Vous souvenir du mois ou de l'année en cours	0	1	2	3
	Gérer votre argent: équilibrer le budget, payer vos factures, gérer les impôts etc.	0	1	2	3
	Vous rappeler de vos rendez-vous	0	1	2	3
	Penser ou vous remémorer	0	1	2	3
	Au cours des deux dernières semaines, avez-vous				
	Eu moins d'intérêt ou de plaisir à faire des choses,des loisirs ou des activités	0	1	2	3
	Répété les mêmes choses encore et encore, comme des questions ou des histoires	0	1	2	3
SECTION 2	***Au cours des deux dernières semaines, avez-vous eu des problèmes pour***				
	Programmer, préparer ou servir les repas	0	1	2	3
	Prendre vos médicaments à la bonne dose ou au bon moment	0	1	2	3
	Marcher ou vous déplacer	0	1	2	3
	Vous laver	0	1	2	3
	Faire vos courses (alimentaires)	0	1	2	3
	Conduire	0	1	2	3
	Faire les tâches ménagères	0	1	2	3
	Au cours des deux dernières semaines, êtes-vous				
	Tombé ou avez-vous trébuché	0	1	2	3
SECTION 3	***Au cours des deux dernières semaines, avez-vous vécu les situations suivantes ?***				
	Se sentir mal, déprimé ou désespéré	0	1	2	3
	Se sentir seul	0	1	2	3
	Rejeter l'aide des autres ou se sentir irrité	0	1	2	3
	Se sentir anxieux, nerveux, tendu, effrayé ou paniqué	0	1	2	3
	Croire que les autres veulent vous voler ou vous faire du mal	0	1	2	3
	Entendre des voix, voir des choses ou parler à des gens qui ne sont pas présents	0	1	2	3
	Avoir trop peu ou trop d'appétit	0	1	2	3
	Tomber endormi, avoir du mal à se lever ou trop dormir	0	1	2	3
	Agir de manière impulsive, sans penser aux conséquences de vos actes	0	1	2	3
	Errer, faire les cent pas ou faire des choses de manière répétitive	0	1	2	3
SECTION 4	***Au cours des deux dernières semaines, avez-vous été préoccupé par :***				
	Votre qualité de vie	0	1	2	3
	Votre avenir financier	0	1	2	3
	Votre santé mentale	0	1	2	3
	Votre santé physique	0	1	2	3
	Total des colonnes				
	**Score**				
